# The prevalence of HIV among tuberculosis patients in Benadir, Somalia. Retrospective multi-center study

**DOI:** 10.1016/j.amsu.2022.103793

**Published:** 2022-05-25

**Authors:** Abdirahman Mohamed Hassan Dirie, Sedat Çolakoğlu, Bashir Mohamud Abdi, Abdiaziz Mohamud Shire, Abdullahi Hassan Abdinur

**Affiliations:** a Mogadishu Somali Turkey Recep Tayyip Erdogan Training and Research Hospital, Pulmonology Department, Mogadishu, Benadir, Somalia; b Hage Institute for Medical Research, Digfer Road, Hodon, Mogadishu, Benadir, Somalia; c Somali Federal Government, Ministry of Health, HIV Care and Treatment Officer, Mogadishu, Benadir, Somalia; d Somali Federal Government, Ministry of Health, National TB Program Manager, Mogadishu, Benadir, Somalia

**Keywords:** Co-infection, Pulmonary TB, Extrapulmonary TB, HIV/TB co-Infection, Tuberculosis

## Abstract

**Introduction:**

In 2020, 8% of TB patients were con-infected with the Human Immuno Deficiency Virus (HIV) and 50% in parts of southern Africa. Due to a lack of available data, we will determine the prevalence of HIV infection among TB patients in the Benadir region.

**Methods:**

This is a record study of the HIV prevalence among TB cases in all Benadir TB centers from July 1, 2019, to June 30, 2020. All those whose HIV status had not been identified and all drug-resistant cases were excluded from this study. Data analysis was performed using IBM SPSS Statistics version 20.

**Results:**

46 out of 3061 (1.5%) patients were HIV/TB co-infected. 63% and 37% were males and females, respectively. 78.2% of HIV-TB co-infected cases were 20–49 years old, which was statistically significant (P = 0.00048). 75.6% of TB/HIV co-infected patients were bacteriologically confirmed cases.

**Conclusion:**

The prevalence of HIV among TB patients in Benadir is very low. This may imply that the prevalence of HIV in the general population is also very low.

## Highlights

• The prevalence of HIV is very low in the TB patients in Somalia.
• The HIV/TB co-infection in Somalia was not the same as in the neighboring countries.
• Young adults have more HIV/TB co-infection.
• HIV/TB co-infection is common in males.
• Generally, TB infection is common in young adults.

## 1 Introduction

Tuberculosis (TB) is a communicable disease that is a major cause of ill health and one of the leading causes of death worldwide. Until the COVID 19 pandemic, TB was the leading cause of death from a single infectious agent [1]. TB can affect anyone, regardless of age or sex, but the highest burden is on adult males, who accounted for 56% of all TB cases in 2020, compared to their adult female counterparts, who accounted for 33%, and children for 11%.

Among all incident cases of TB, 8% were people living with the Human Immuno Deficiency Virus (HIV). The proportion of TB cases co-infected with HIV was highest in countries in the African region, exceeding 50% in parts of southern Africa [2]. A 25% HIV prevalence among adult TB patients was reported in Port Harcourt, Nigeria [3].

HIV infection has effects on the pathogenesis of TB infection because of the suppressed immunity of HIV patients, which increases the possibility of latent TB reactivation and progression from TB infection to TB disease [4]. The co-infection of HIV-1/TB synergistically decreased the viability of macrophages and increased levels of pro-inflammatory cytokines, and this seemed to be specific to M. tuberculosis rather than other species of mycobacteria [5]. On the other hand, TB is one of the most common opportunistic infections in HIV patients and is the leading cause of death in HIV-infected people, and HIV infection is one of the most common risk factors for developing active TB disease from a latent TB infection [6].

A WHO report in 2019 estimated that incident TB cases were attributable to health-related risk factors. About 2.2 million cases were attributable to under-nutrition, 0.76 million due to HIV infection, 0.72 million due to alcohol use disorders, 0.70 million due to smoking, and 0.35 million due to diabetes, which makes HIV the second most common risk factor for TB infection [7]. Many studies state that HIV/TB co-infection leads to the deterioration of both diseases, as HIV leads to the reactivation of latent TB. TB also leads to the rapid progression of HIV infection, so this increases the mortality of both diseases [8].

Globally, in 2019, an estimated 10.0 million people fell ill with TB, and there were 1.2 million TB deaths among HIV-negative people and an additional 208,000 deaths among HIV-positive people. TB is considered the most common cause of death in HIV-positive adult patients in developing countries, though it is a preventable disease [9].

According to the World Health Organization (WHO), TB/HIV co-infection has been found to reduce the effectiveness of directly observed therapy (DOT) treatment of TB [10]. Improving case detection of TB/HIV co-infection potentially leads to early treatment of both conditions and can impact positively on treatment outcomes [11].

Due to a lack of available data, the prevalence of TB/HIV co-infection among TB patients in Somalia is unknown. In this research, we want to determine the prevalence of HIV infection among TB patients in Benadir, the most populated province in Somalia, which includes the capital city of the country.

## 2 Materials and methods

This is a cross-sectional, retrospective record study of the HIV prevalence among TB cases that were recruited in all Benadir TB centers. This study was conducted on TB cases documented from July 1, 2019, to June 30, 2020. All TB patients with known HIV status were included in this study. TB cases were defined as cases with positive Mycobacterium tuberculosis (MTB) by Gene XpertMTB/RIF or treated by radiological and clinical findings or extrapulmonary TB.

The socio-demographic and clinical data, including gender, age, address, HIV status, site of TB infection, and outcome, were retrieved from the Register Book of the TB centers. All those whose HIV status has not been documented in the TB register book and all DR, MDR, and XDR patients were excluded from this study.

Data analysis was performed using IBM SPSS Statistics version 20 (IBM Corp. @Copyright IBM Corporation and its licensors 1989, 2011, Trademarks of Oracle and its affiliates). The prevalence of HIV infection among the TB patients was estimated by using the confidence limits of the P-value (P = 0.05). This work has been reported in line with the STROCSS criteria [12].

## 3 Ethical Considerations

The study protocol was cleared by the MOGADISHU SOMALI TURKEY, RECEP TAYYIP ERDOGAN TRAINING AND RESEARCH HOSPITAL, Research Ethics Committee Approval Ref No: MSTH/4999/284, on 12/12/2020 and the Somali Ministry of Health, National TB Program, Ref No: FMOH/NP/009/2022, on January 10, 2022.

## 4 Results

**Fig. 1:**
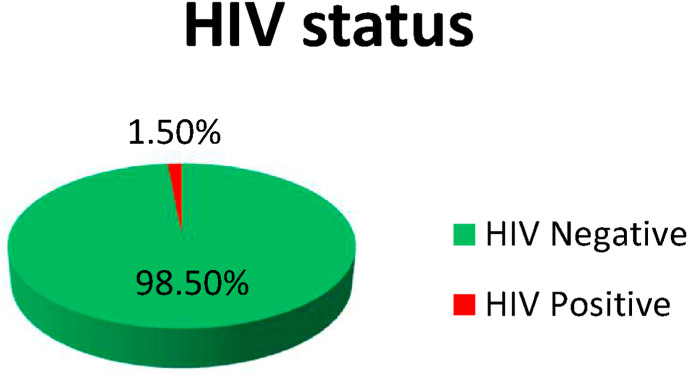
HIV status of all TB patients.

Overall, 3165 patients were collected and almost 3061 (96.7%) were eligible (treated TB cases with known HIV status), while 79 cases were missing data, 14 cases were transferred from other TB centers outside of Benadir Province without documented HIV status, and 11 cases had HIV tests that were not available, and all those groups were excluded from this study. The number of patients with HIV/TB co-infection was 46 patients (1.5%) out of 3061, while the other 3015 (98.5%) out of 3061 were HIV-negative cases (Fig. 1).

**Fig. 2:**
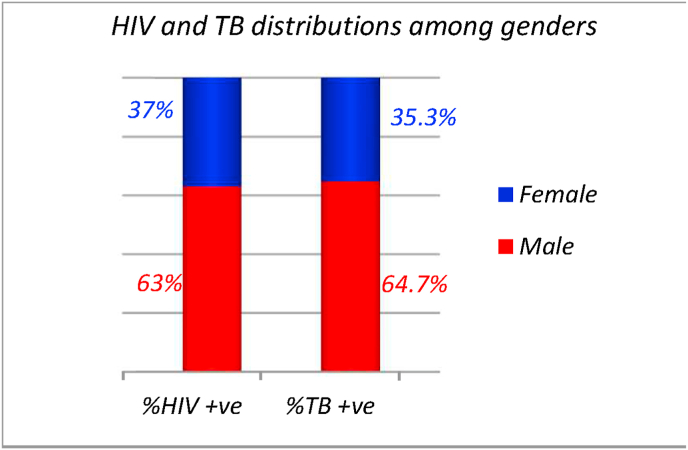
HIV and TB percentages among both genders. Red chart shows male portion while blue charts states female portion. (For interpretation of the references to colour in this figure legend, the reader is referred to the Web version of this article.)

The majority of TB patients in our study were males 1981 (64.7%) while female patients were 1080 (35.3%) (Fig. 2). Among the 46 HIV positive patients, 29 (63%) were males, while 17 (37%) were females, which was not statistically significant (P = 0.46, a = 0.05) (Fig. 2).

**Fig. 3:**
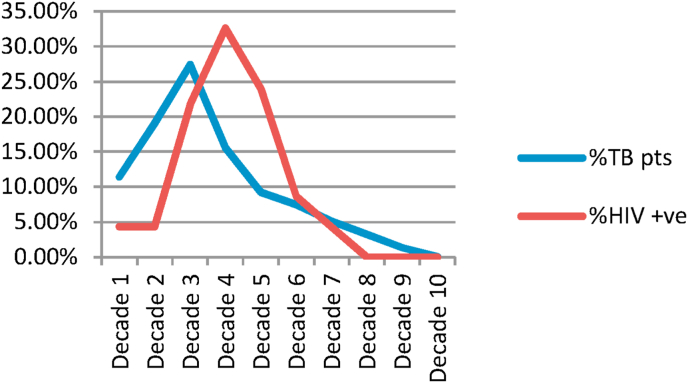
Red chart shows age group distribution of HIV positive patients while blue chart states age group distribution of TB positive patients. (For interpretation of the references to colour in this figure legend, the reader is referred to the Web version of this article.)

The majority of TB patients (73.4%) were young adults and children. Approximately two thirds (62.1%) of our TB cases were in the age group between 10 and 39 years old (Fig. 3). The mean, median, and mode of the age of all TB patients were 30.2, 26, and 30, respectively, with the average age of males being 30.9 years and females being 28.8 years. On the other hand, the mean, median, and mode of the age of HIV-TB co-infected cases were 34.2, 35, and 30 years old, respectively, with a mean age of 34.6 years for males and 33.9 years for females.

Among the HIV-TB coinfected cases, most of them (78.2%, N = 36) were young adults with an age group between 20 and 49 years old, and the third decade was the most commonly affected age group, constituting about 32.6% (N = 15) of all HIV-TB co-infected patients, which was statistically significant by Pearson's Chi-Square with a P-value of = 0.00048 (a = 0.05) (Fig. 3).

The cases with sputum positive for mycobacterium tuberculosis were 1641 (53.6%), extrapulmonary 873 (28.5%), and cases treated by radiological and clinical findings were 509 (16.6%), and this was statistically significant relation between bacteriologically confirmed HIV cases and their HIV co-infection (P = 0.035). On the other hand, the majority of the TB/HIV co-infection cases that had a positive GeneXpert for Mycobacterium tuberculosis were 34 cases (75.6%), and extra-pulmonary TB cases were 4 (8.7%), while other cases that were treated by radiological and clinical diagnosis were 8 (17.4%) (Table 1). The anti-retroviral therapy status of the TB/HIV patients was not available in this study, so they are not included in our study.

**Table 1:** Showing percentage of TB smear positive, radiologically treated and Extra-pulmonary among HIV/co-infected patients.

	Frequency	Percent
**Gene Sensitive**	34	73.9%
**Negative**	5	10.9%
**Not Available**	2	4.3%
**Extrapulmonary**	4	8.7%
**Missing**	1	2.2%
**Total**	46	100%

## 5 Discussion

The prevalence of HIV infection among the TB cases that were treated in the Benadir TB centers was 1.5%, which is consistent with the prevalence that has been reported in China (0.5%) [13], but it was more than five times lower than that reported in the WHO 2020 Global TB Report, where the prevalence of HIV/TB co-infection was 8% of all TB patients worldwide and it exceeded 50% in parts of southern Africa, making the prevalence of HIV among TB patients in our country 33 times less than those reported in some southern African countries. The overall prevalence of HIV among TB patients in northeast Ethiopia was reported to be 40.4%, but only 11.8% among Afar ethnics [14]. Our study shows approximately 27 times lower HIV prevalence among TB patients than that in the Northeast of Ethiopia, the neighboring country to Somalia.

A countrywide cross-sectional survey which was conducted in Zambia in 2013/2014 showed that 23.8% of TB patients were HIV positive, making the prevalence of HIV among TB patients in our study 16 times lower than that reported in Zambia [15]. In Eastern India, a cross-sectional record analysis study covering a 10-year period stated that the HIV prevalence among TB patients was reaching up to 12.3% [16], making our results eight times less than what has been reported in Eastern India. Our study shows that the prevalence of HIV/TB co-infection is higher in males than females, which was not statistically significant at 63% (P = 0.81, a = 0.05).

Surveys in Africa, Asia, and the Pacific have indicated that the HIV prevalence among TB patients is much higher than that observed in the general population [17]. This implies that HIV prevalence in the general population of Somalia is very low when compared to high TB burden countries in the region because people living with HIV infection are more prone to contracting TB infection than HIV-negative people. TB/HIV co-infected individuals who fall sick are more likely to go to the health facility earlier than others due to the severity of symptoms, their awareness of their low immunity, and their susceptibility to TB infection, so they are potentially diagnosed in the earlier stages of the disease process. In addition to that, TB screening programs are recommended among HIV patients, so that is another factor that can increase the chance of detection of new TB cases in the HIV population during anti-retroviral visits (ART).

The reason for the low HIV prevalence among TB patients in Benadir is probably due to the low HIV prevalence in the general population, which might be a result of religious, cultural, and constitutional restrictions on multiple sex partners and sex-oriented businesses. A community survey in Zambia analyzed a sample of 2610 men and women and 445 community leaders in 36 districts, and the findings showed that having multiple partners is the strongest predictor of the perceived risk of HIV [18]. Also, Eastern Indian cross-sectional record analysis showed that 86% of the HIV-TB co-infected cases had histories of heterosexual relationships with more than one partner [19].

TB-HIV co-infected patients are known to have poorer treatment outcomes, and therefore, those patients who do not seek care or do not have access to medical care are more likely to die earlier than other infected cases in the community. A countrywide cross-sectional survey and extensive research covering all provinces of the country are required for the detection, tracing, and management of HIV/TB co-infections at the community level. In our study, we found that TB/HIV co-infection was more common in the age groups that TB infection affects most of the time, but the mean age was slightly higher in TB/HIV co-infected patients than in HIV-negative TB-infected patients. It should be noted that all cases we have studied in our research were patients treated as TB patients irrespective of their site of infection, sputum culture results, AFB, and GeneXpert status findings.

All diagnostic procedures for TB infections were applied to our patients, including chest X-rays, chest CT scans, three consecutive sputum collections for Acid Fast Bacilli (AFB), and the GeneXpert MBT/RIF assay, which is the most accurate diagnostic tool, and it was compulsory for all suspected TB patients to rule out drug-resistant TB infection. To our knowledge, there is no research published about the prevalence of HIV among TB patients in Somalia. So, we recommend a countrywide research project to understand the burden of HIV-TB co-infection in all provinces to detect and treat TB/HIV cases early.

## 6 Conclusions

In conclusion, the prevalence of HIV among TB patients in Benadir province of Somalia, including the capital city of Mogadishu, is very low compared to the neighboring countries and other high-TB burden countries. We found that female patients have significantly lower HIV/TB co-infection as they also have a lower TB prevalence compared to male patients, but statistically it was not significant. The majority of HIV/TB co-infected patients were young adults. This data suggests that the prevalence of HIV among the Somali population is probably very low, probably due to religious, cultural, and constitutional restrictions on multiple sex partners and sex-oriented businesses. However, a population-based survey study about the prevalence of HIV/TB co-infection is needed.

## Ethical approval

The Research Ethics Committee of our hospital and the Ministry of Health/NTP have approved.

## Sources of funding

No sources of funding to declare.

## Provenance and peer review

Not commissioned, externally peer-reviewed.

## Sources of funding for your research

No sources of funding to declare.

## Ethical approval

Research Ethics Committee of our hospital and Ministry of Health/NTP has approved.

## Consent

This was retrospective study so no consent.

## Author contribution

Abdirahman Mohamed Hassan Dirie: designing of research plan, data collection, data analysis, literature review, writing of results, discussion, introduction and conclusion.Sedat Çolakoğlu: designing and research planning and facilitated getting permision from research committee of our hospital Bashir Mohamud Abdi: played an important role in data collection Abdiaziz Mohamud Shire: Data collection and facilitated to get permision from ministry of health Abdullahi Hassan Abdinur; took part in discussion and introduction writing.

## Registration of research studies

1. Name of the registry: Not applicable
2. Unique Identifying number or registration ID: Not applicable
3. Hyperlink to your specific registration (must be publicly accessible and will be checked): Not applicable

## Guarantor

Abdirahman Mohamed Hassan Dirie.

Sedat Çolakoğlu.

Abdiaziz Mohamud Shire.

## Declaration of competing interest

Authors have no financial or personal conflict of interest.
